# Digital measurement of ocular microtremor in Parkinson’s Disease: Analytical and clinical validation

**DOI:** 10.1371/journal.pdig.0001439

**Published:** 2026-06-18

**Authors:** Lisa Graham, Rodrigo Vitorio, Patrick Tait, Richard Walker, Alan Godfrey, Rosie Morris, Samuel Stuart

**Affiliations:** 1 Department of Sport, Exercise and Rehabilitation, Northumbria University, Newcastle, United Kingdom‌‌; 2 Gateshead Health NHS foundation trust, Gateshead, United Kingdom; 3 Northumbria Healthcare NHS foundation trust, North Shields, United Kingdom; 4 Department of Computer and Information Sciences, Northumbria University, United Kingdom; 5 Oregon Health & Science University, Portland, Oregon, United States of America; Drexel University, UNITED STATES OF AMERICA

## Abstract

Ocular microtremor (OMT) is an involuntary fixational eye movement linked to brainstem activity. OMT is thought to have a mean frequency range of 70–90 Hz in healthy adults. Previous research suggests OMT may be reduced in neurological diseases like Parkinson’s Disease. Historically, OMT has been measured invasively in specialist laboratories using lengthy and expensive protocols. Developments now allow for OMT measurement quickly and non-invasively using hand-held technology (i.e., iTremor ONE). This pilot study aimed to examine the analytical and clinical validation of OMT measurement via the iTremor ONE in people with Parkinson’s Disease (PwPD). 33 PwPD and 31 age matched healthy controls participated in this study. For analytical validation, 22 PwPD completed a test re-test reliability assessment of OMT measurement, assessed using interclass correlation coefficients (ICC). For clinical validation, OMT frequency in PwPD (n = 33) was compared to controls. Correlations were explored with demographics and clinical scales. Additionally, 24 PwPD were tested ‘OFF’ (12hr withdrawal) and ‘ON’ their anti-Parkinson’s (dopaminergic) medication to compare OMT response to a known intervention. The iTremor ONE demonstrated excellent test-retest reliability (ICC > 0.9) for measuring OMT frequency in PwPD. Mean OMT frequency was significantly lower in PwPD (63.78 ± 4.82 Hz) compared to controls (69.44 ± 6.47 Hz, p < .001), with good discriminative ability (AUC 0.75-0.77). OMT frequency correlated with age in both groups and with specific motor features (speech, facial expression, gait) in PwPD. No significant differences in OMT frequency were observed between ‘OFF’ and ‘ON’ dopaminergic medication states. This is the first study to demonstrate that a non-invasive hand-held device can reliably measure OMT in PwPD and presents OMT analytical and clinical validation evidence. OMT frequency may provide a supporting measure for diagnosis or screening. Further research is required to understand the neural mechanisms underpinning OMT in PwPD and the role it could play in clinical practice.

## Author summary

Parkinson’s disease (PD) is one of the fastest growing neurological conditions globally, making early and accurate diagnosis increasingly important. Current assessment methods rely heavily on clinical observation, which can be subjective and may miss subtle early changes. We investigated whether a microscopic involuntary eye movement called ocular microtremor (OMT) could serve as an objective digital eye movement outcome measure for PD. Historically, measuring OMT required invasive, lengthy procedures where patients had their eyes held open for extended periods with probes placed directly on the eyeball, making them impractical for routine clinical use. We tested a new handheld device that measures OMT non-invasively for the first time in just three seconds using safe laser technology. We found the device gave highly reliable measurements and could distinguish between people with Parkinson’s disease and healthy older adults with good accuracy. Interestingly, OMT related to specific symptoms like facial expression difficulties and walking problems but did not respond to PD medication. This suggests OMT may reflect brain chemistry beyond the dopamine system targeted by standard treatments, potentially involving other chemical messengers like acetylcholine. While larger studies are needed to confirm these findings, this quick, comfortable test could potentially complement existing tools for earlier diagnosis or disease monitoring in the condition.

## 1 Introduction

Eye movements have long been considered as a window into the brain, offering important information about neurological activity and eye health [1,2]. Examination of eye movements is a key tool in neurological assessment as eye movements can be attributed to specific brain regions and their function [3–5]. Both voluntary and involuntary eye movements have been identified as promising biomarkers in neurological injury and disease and may provide a novel way to predict and track disease [6–10]. Current methodologies for the collection of eye movement data predominantly involve techniques which are highly subjective and often require lengthy protocols and invasive techniques [11]. As a result, there is a need for a quick, reliable, and non-invasive objective measurement tool to assess eye movements, including involuntary fixational eye movements.

One such fixational eye movement is ocular microtremor (OMT), a subtle, microscopic eye movement that is present in everyone at all times [12]. OMT has gained some attention as a potential diagnostic and clinical tool in ophthalmology and neurology [13]. It is distinct from larger more noticeable eye movements, such as saccades or smooth pursuit, and could potentially act as a more sensitive marker for neurological functioning, due to the complex neural mechanisms that underlie fixation [14]. It is an involuntary movement that has been reported to range from 70-150Hz (oscillates at 70–150 times per second [15]), in healthy subjects and cannot be seen by the naked eye, requiring specialised techniques to be quantified [16]. There is no gold standard technique for measurement of OMT, meaning that the application of OMT measurement protocols can vary as the microscopic movement is difficult to detect (e.g., video based eye-tracking systems don’t possess the sensitivity required to detect OMT) [17]. Consequently, this limits generalisability and interpretation of underlying deficits [18]. Existing methods rely on invasive tools, such as the piezoelectric strain gauge technique, a probe is placed directly onto the eyeball, accelerometer fixed contact lenses, or eyelid fixed accelerometers which have several limitations (e.g., invasive nature, duration of protocols, need for specialist laboratory space and staff, and expense of equipment) [18]. Additionally, strain gauges or other techniques require the eyes to be anaesthetised [12,19–25], possess high interobserver variability, require a highly trained operator, and cause discomfort and eye dryness. It also relies on constant uninterrupted contact with the sclera meaning patients had their eye held open for up to 60minutes at a time, and there is an unknown loading effect of the probe, making validation of new techniques against this one undesirable [17,18,26]. Overall, invasive methods have limited application as a clinical tool for objective OMT as a biomarker. Given the previously demonstrated clinical utility, there is a need for a portable and non-invasive solution for measuring OMT. Further studies and advancements in OMT measurement techniques may lead to its potential integration into clinical practice for diagnostic and monitoring purposes in the future.

To date, there is little research on OMT, likely due to the lack of a quick, non-invasive measurement technique. As such, the underlying mechanisms of this subtle eye movement are not fully established. OMT has been postulated to link to brainstem activity [16,22,27]. Previous research has observed abnormal OMT characteristics in neurological conditions, such as Parkinson’s Disease (PD) and Multiple Sclerosis [18,24,28,29]. It is thought that OMT originates from neural activity involving brainstem structures that excites the ocular motor nerves and causes the eye muscles to tremble [16,27,23]. However, the specific brainstem nuclei, pathways, and neurotransmitter systems involved remain uncertain.

Previous work has shown altered OMT in PD [29]. PD is the fastest growing neurological disease in the world [30], with an estimated prevalence of 8.5 million globally [31]. While the hallmark motor features of PD result from dopaminergic cell loss in the substantia nigra pars compacta, pathological changes in PD affect brainstem nuclei early in the disease course [32]. The pathophysiology extends to multiple brainstem nuclei, including noradrenergic (locus coeruleus), serotonergic (raphe nuclei), and cholinergic (pedunculopontine nucleus) systems that have roles in various aspects of motor and non-motor function [33]. In PD, there are widespread neural mechanisms that underpin deficits in eye movement control. Given the hypothesized brainstem involvement in OMT generation and the documented brainstem pathology in PD, OMT abnormalities may reflect dysfunction in these neural systems. Despite evidence of altered OMT in PD using invasive measurement methods and the hypothesised link to brainstem pathology, no study has yet examined OMT using a non-invasive, portable technique in a clinical PD population. This represents a critical gap in the literature, as the absence of a non-invasive and practical measurement tool has prevented OMT from being evaluated as a clinically viable biomarker in PD.

**Fig 1:**
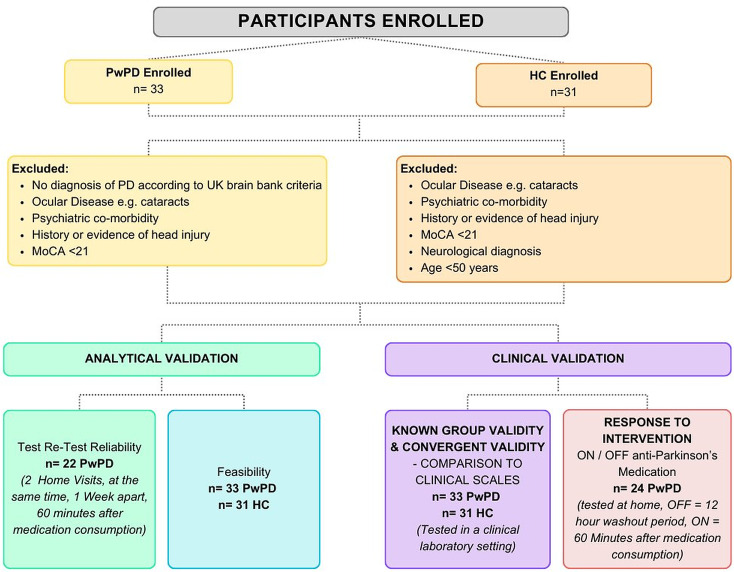
Overview of Study Design and Participant Inclusion for Analytical and Clinical Validation‌‌.

Recent developments have led to the ability to record OMT with non-invasive and portable technology (i.e., The iTremor ONE, Head Diagnostics Ltd., Dublin, Ireland), which could allow relatively fast OMT measurement in various locations. This device uses laser technology to measure OMT and offers a high resolution non-invasive, compact, and portable technique for obtaining OMT data [34,35]. This method has been proven to be feasible and overcomes many of the current limitations associated with other more invasive techniques [36]. However, this new device has yet to undergo analytical and clinical validation to determine whether it is accurate, reliable, and sensitive to clinical groups of interest, such as PD. Therefore, this pilot study aimed to examine analytical and clinical validation of OMT measurement via the iTremor ONE in PD. See Fig 1 for a study overview.

*Analytical Validation:* (1) Investigate the test-retest reliability of the iTremor ONE in measuring OMT frequency (Hz) in people with PD (PwPD); (2) Investigate the feasibility of measuring OMT using hand-held technology in a real-world clinical setting in PwPD. We hypothesised that OMT measurement with the iTremor ONE would be reliable and feasible for use with PwPD.

*Clinical Validation:* (1) Compare OMT frequency (Hz) in PwPD compared to age-matched healthy controls (HC); (2) Explore relationships between OMT measurement in PwPD and clinically relevant outcomes (e.g., disease severity); and (3) Examine the effect on OMT of dopaminergic medication in PD. We hypothesized that OMT measurements would be different between PwPD and HCs, would associate with clinical measures of disease, and be influenced by dopaminergic medications.

## 2 Methods

Our full protocol has been published previously, please consult this for further details (https://doi.org/10.1371/journal.pone.0313452) [37].

### 2.1 Ethics statement

A Northumbria University research ethics committee granted ethical approval (Project No. 0034). The trial has been reviewed and registered with Clinicaltrials.gov (NCT06051877). This study also received NHS Research Ethics Committee approval (REF: 23/WM/0004), and Medicines and Healthcare products Regulatory Agency (MHRA) letter of no objection (REF: CI/2023/0031/GB). Written consent was obtained from all participants prior to any study activity.

### 2.2 Inclusion and exclusion criteria

PD participants were recruited from the Northumbria Healthcare NHS Foundation Trust movement disorders clinic and from local PD support groups. PwPD were included if they had a diagnosis of PD according to UK brain bank criteria [38,39]. Age-matched HCs were recruited from family members of PwPD, as well as from Northumbria university staff and were aged 50 years old or above. Participants were excluded if they had any psychiatric co-morbidity, a history or evidence of head injury or ocular disease (such as cataracts) or a clinical diagnosis of dementia or other severe cognitive impairment (measured using the MoCA with a score of <21).

### 2.3 Equipment

**Fig 2:**
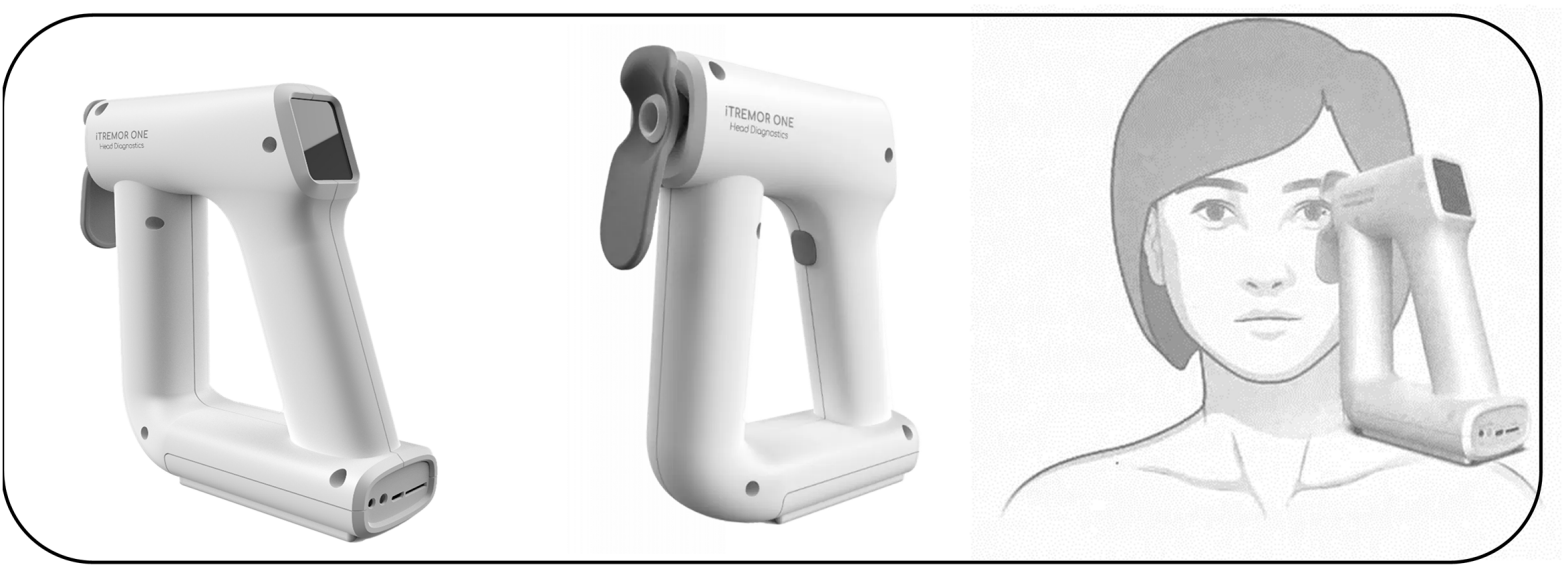
iTremor ONE device and placement.

OMT was measured with the iTremor ONE (Head Diagnostics Ltd., Dublin, Ireland – see Fig 2, software version 0.19), which takes three-seconds to record OMT in each eye after alignment. The iTremor ONE used a method based on measuring angular displacement determined from laser speckle correlation of images recorded in the Fourier plane of a lens to measure OMT [34]. The iTremor ONE segregates OMT from other eye movements, hand movements of the user holding the iTremor ONE and subtle head movements of participants with filtering (e.g., band-pass).

### 2.4 Analytical validation in PD

#### 2.4.1 Participants and environment.

To assess the test-retest reliability of OMT measurement in PwPD, 22 PwPD participated in two home-based assessments performed exactly one week apart, at the same time of day (within 30 minutes) and in the same location in the home. At the first visit, demographic data was collected, including age, gender, education level, falls history and medications.

#### 2.4.2 Clinical assessment.

In both visits, disease severity was assessed in the “ON” state 1-hour following Levodopa medication intake. Disease severity was assessed using the Movement Disorder Society Unified Parkinson’s disease Rating scale part III (UPDRS-III [40]).

#### 2.4.3 OMT measurement procedures.

For the OMT measurement, participants were asked to sit in a comfortable, high-backed chair, and any curtains/blinds in the room were closed to control for differences in light (i.e., dimmed light). If the participant wore glasses or contact lenses, they were asked to remove these for the duration of the recordings. OMT measurement involves a low-level laser directed onto the surface of the eye, specifically the sclera, and lasting approximately 3s. For the duration of the recording, participants were asked to look straight ahead at a target positioned on the wall directly in-front of them at eye level, ensuring to maintain a relaxed gaze, so as to not strain the eye muscles. If the participant blinked, the reading was repeated. A frequency reading in Hz was provided on the display screen. Three readings were taken for each eye that were averaged. A single iTremor ONE was used within this study, with the same operator for every measurement.

### 2.5 Clinical validation in PwPD

#### 2.5.1 Participants.

Clinical validation involved OMT data from 33 PwPD in total. Of these, 22 PwPD were assessed in the home environment as part of the analytical validation (second visit data used for clinical validation) and an additional 11 PwPD were assessed in the laboratory, giving a total of 33 PwPD. 31 HCs were assessed in the laboratory. To test in the ‘OFF’ and then ‘ON’ medication state (1-hour after Levodopa intake) 24 of the overall 33 PwPD were assessed in the home environment.

#### 2.5.2 Clinical assessment.

Age, gender, education (years), fear of falling (i.e., Falls Efficacy Scale (international version) (FES-I)), visual acuity (LogMar) and contrast sensitivity (LogCS) (measured via standard eyecharts) were collected for all participants. The following were collected specifically in PwPD: disease duration (years since diagnosis), disease severity (via MDS-UPDRS part III and Hoehn and Yahr (H&Y) stage), freezing of gait status (via freezing of gait questionnaire – FOGQ), levodopa equivalent daily dose (LEDD) and hours since last dose [41].

#### 2.5.3 OMT measurement procedures.

All OMT readings in clinical validation testing were carried out in the same manner as per analytical validation, with participants seated in a high-backed chair, looking straight ahead at a target at eye level. Room lighting was controlled consistently across all scenarios. Testing was conducted in a quiet environment, free from noise and interruption. Three measures per eye were averaged. All assessments were conducted by the same trained operator using a single iTremor ONE device to ensure measurement consistency.

#### 2.5.4 Response to dopaminergic medication.

To assess the effect of anti-Parkinson’s medication (levodopa) on OMT, 24 PwPD must have not taken dopaminergic medication for a minimum of 12 hours prior to the testing visit. In this ‘OFF’ state OMT frequency was assessed. Participants then took their morning dose of medication, waited 60 minutes, and their OMT frequency was assessed again.

### 2.6 Data analysis

Data was analysed using SPSS (v28, IBM Inc, IL, USA). Normal data distribution was confirmed using Kolmogorov-Smirnov tests and Shapiro-Wilk tests (all Shapiro-Wilk p > .05). Demographics were reported using descriptive statistics. OMT frequency outcomes were assessed as an average overall (for both eyes) and then for left and right eyes separately. Where valid OMT readings could not be obtained, data were treated as missing and excluded from analysis. All analyses used available case analysis. A summary of missing cases has been included as Supplementary Material (see S1 Table.) to provide full transparency. Given the exploratory nature of this study, we did not control for multiple comparison corrections (e.g., Bonferroni), which may be overly conservative in this preliminary investigation [42–44].

#### 2.6.1 Analytical validation.

*Reliability* Absolute agreement between OMT measurement in visit 1 and 2 was assessed using intra-class correlations (ICC_2,1_). ICCs were interpreted as; poor <0.5, moderate 0.50-0.75, good 0.75-0.90 and excellent >0.90 [45]. Additionally, Pearson’s correlations and Bland-Altman graphs (given in Online Resource 1, S1 Fig.), with mean differences and limits of agreement, compared OMT between the two sessions.

*Feasibility* Feasibility outcomes including number of adverse events and device issues were descriptively reported.

#### 2.6.2 Clinical validation.

*Known groups validity* To examine whether OMT can differentiate PwPD from HCs, an ANOVA was used, with group (PwPD vs. HCs) as a between-subject factor. Tukey post-hoc analysis was performed to determine individual group differences in OMT measures. Receiver operating characteristic (ROC) curve analysis tested the performance of OMT frequency from each eye in discriminating PwPD from HCs. The area under the curve (AUC), sensitivity and specificity were calculated. AUC was interpreted as; outstanding >0.9, excellent 0.8-0.9, good 0.7-0.8, fair 0.6-0.7, fail <0.5 [46]. The optimal cut off point for each outcome measure was determined as the point closest to corner (0,1) with a false positive rate = 0%, sensitivity 100% in the ROC plane [47].

*Convergent validity* OMT frequencies were correlated with demographic and clinical measures. Normal distributions were determined, and the appropriate tests were performed (Pearson’s where normally distributed/ Spearman’s where not normally distributed).

*Response to a known intervention* To examine whether OMT reduces when in the ‘OFF’ medication state in PD, paired t-tests were performed with medication-state groups (‘ON’ vs ‘OFF’ medication).

## 3 Results

### 3.1 Participants

*Analytical validation cohort* Table 1 shows the participant demographic characteristics for cohort included participants (PwPD n = 22). On average, PwPD had mild to moderate disease severity, and preserved global cognition (MoCA ~ 27).

**Table 1:** Participant Demographics, Clinical and OMT Outcomes.

	Analytical Validation			Clinical Validation					
	*Visit 1*	*Visit 2*		*Group Differentiation and Comparison to Clinical Rating Scales*			*Response to Known Intervention*		
	PwPD (n = 22)		ICC^2,1^	HC (n = 31)	PwPD (n = 33)	*p*	*PwPD OFF (n = 24)*	*PwPD ON (n = 24)*	*p*
**Age (Years)**	68.82 (7.74)	–	–	67.32 (9.92)	69.06 (7.56)	.432	67.37 (9.65)	**–**	–
**Sex**	11M/ 11F	–	–	12M/ 19F	19M/ 14F	.135	13M/ 11F	–	–
**Education (Years)**	13.64 (3.89)	–	–	16.01 (4.34)	13.73 (3.65)	**.023**	14.24 (3.76)	–	–
**FES-I**	27.69 (10.03)	–	–	18.92 (2.41)	28.31 (11.14)	**<.001**	26.53 (9.63)	–	–
**Cognition**									
**MoCA**	27.45 (2.24)		–	26.45 (2.06)	27.00 (1.98)	.283	26.33 (2.57)	27.57 (2.06)	**0.03**
**TMT A (seconds)**	–	–	–	28.04 (10.69)	41.38 (23.09)	**.007**	**–**	**–**	**–**
**TMT B (seconds)**	–	–	–	60.08 22.77)	73.71 (44.86)	.163	**–**	**–**	**–**
**CLOX1**	–	–	–	13.07 (1.69)	12.71 (2.27)	.640	–	–	–
**CLOX2**	–	–	–	14.21 (0.80)	13.93 (1.44)	.522	–	–	–
**JLO**	–	–	–	21.27 (5.50)	20.63 (5.93)	.721	–	–	–
**Forward digit span**	–	–	–	6.73 (3.83)	6.61 (1.26)	.869	–	–	–
**Vision**									
**Visual Acuity**	–	–	–	0.16 (0.16)	0.21 (0.13)	.277	–	–	–
**Contrast Sensitivity**	–	–	–	1.59 (0.14)	1.40 (0.32)	**.010**	–	–	–
**Clinical**									
**Disease Duration (Years)**	4.64 (3.79)	–	–	–	5.08 (3.75)	–	4.54 (3.68)	–	–
**UPDRS III**	35.18 (15.28)	–	–	–	35.94 (17.71)	–	36.96 (15.29)	31.91 (15.38)	0.22
**Most affected side**	7R, 10L, 5B	–	–	–	8R/ 18L/ 6B	–	9R/ 15L/ 0B	7R/ 13L/ 4B	–
**H&Y Stage**	5I, 9II, 7III,1 IV	–	–	–	6 I/ 16 II/ 9 III/ 2 IV	–	5 I/ 14 II/ 5 III	5 I/ 11 II/ 7 III	–
**nFOGQ**	7.05 (8.64)	–	–	–	6.24 (8.06)	–	5.29 (7.24)	–	–
**LEDD**	397.34 (228.24)	–	–	–	361.50 (243.26)	–	381.48 (190.23)	–	–
**Hrs since Ldopa**	1	1	–	–	2.11 (0.10)	–	1.41 (0.88)	15.00 (3.75)	–
**OMT Measures**									
OMT Right eye (Hz)	64.85 (4.52)	65.98 (4.71)	.94 (.86 -.98)	69.65 (7.33)	63.98 (5.38)	**<.001 (0.88)**	66.15 (4.58)	65.55 (4.62)	.282
OMT Left eye (Hz)	63.93 (4.99)	65.62 (4.19)	.93 (.82 -.97)	69.24 (5.75)	63.58 (4.58)	**<.001 (1.09)**	65.16 (4.74)	64.56 (4.87)	.193
OMT Both eyes (Hz)	63.92 (4.90)	65.64 (4.24)	.92 (.81 -.967)	69.44 (6.47)	63.78 (4.82)	**<.001 (0.99)**	65.65 (4.37)	65.05 (4.61)	.158

SD = Standard deviation, PwPD = People with Parkinson’s Disease, ICC = Intraclass Corelation Coefficient, HC = Healthy Control, FES-I = Falls Efficacy Scale – Internation, MoCA = Montreal Cognitive Assessment, TMTA/B = Trail making task, CLOX1/2 = Royall’s clock drawing task, JLO = Judgment of Line Orientation task, UPDRS = MDS Unified Parkinson’s Disease Rating Scale III, H&Y = Hohen & Yahr Stage, nFOGQ = new Freezing of Gait Questionnaire, Hrs = Hours, Ldopa = Levodopa, OMT = ocular microtremor, r = correlation value, p = significance value (p value reported with Cohen’s d in parentheses for primary OMT outcome measures).

*Clinical validation cohort* Table 1 also shows the participant characteristics for cohort included participants (PwPD n = 33 and HC n = 31). On average, PwPD were aged matched to the control cohort and had mild to moderate disease severity and preserved global cognition (MoCA ~ 27).

*Response to known intervention cohort* Table 1 also shows the participant characteristics for cohort included participants (PwPD n = 24). On average, PwPD had mild to moderate disease severity and preserved global cognition when ‘OFF’ and ‘ON’ medication ((MoCA ~ 27 and ~26, respectively).

### 3.2 Analytical validation of OMT measurement

#### 3.2.1 Test retest reliability.

Table 1 shows the OMT frequency (Hz) for PwPD at the home visits demonstrating excellent agreement between the OMT readings (all ICC > 0.90). On average, there was little difference between the readings from the first and second visits (mean difference 1.14 Hz), with small Limits of agreement (LoA% 6.6) in the right eye, and similarly in the left eye (mean difference 1.70, LoA% 7.4), and both eyes (mean difference 1.73, LoA% 7.5) (See S1 Fig).

#### 3.2.2 Feasibility.

No adverse events or device issues were recorded during this study. On rare occasions an OMT measurement was repeated by the examiner to obtain the reading if the iTremor ONE reported inability to take OMT reading (typical circumstances included eye blink, obstruction from eye lashes or eye lids, and change of gaze direction during the measurement) but it was always possible to obtain the required number of recordings within a timely manner in the session.

### 3.3 Clinical validation of OMT measurement

#### 3.3.1 Known group validity.

**Fig 3:**
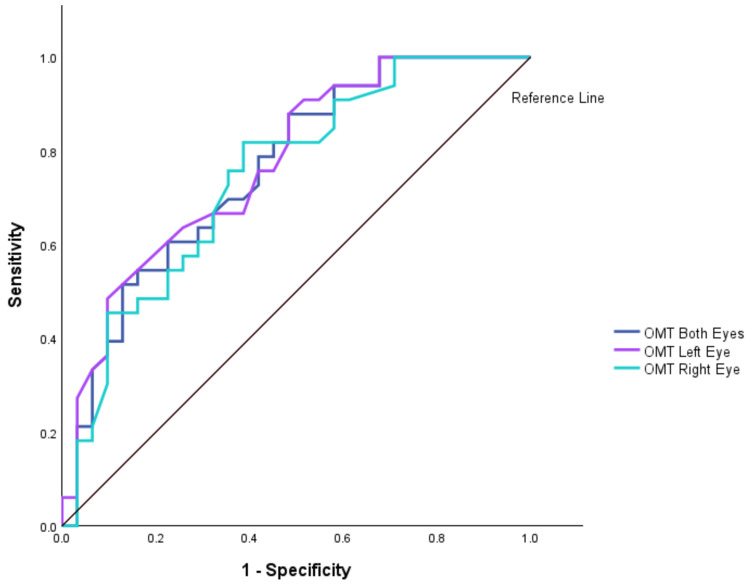
Box and Scatter plots for distribution of mean OMT frequency between PwPD and HC.

Table 1 demonstrates there were significant differences between PwPD and HC in OMT measurement for the right, left and both eyes. As shown in Fig 3, HC displayed higher mean OMT frequency (right eye 69.65Hz SD = 7.33, left eye 69.24 SD = 5.75, both eyes 69.44 SD = 6.47) while PwPD displayed lower mean OMT frequency (right eye 63.98Hz SD = 5.38, left eye 63.58 SD = 4.58, both eyes 63.78 SD = 4.82). Mean OMT frequency (Hz) was significantly different between PwPD and HCs for right, left and both eyes (*p =* < .001, Table 1). Effect size analysis revealed large effects for all OMT measures (Cohen’s d = 0.88, 1.09, and 0.99 for right, left and both eyes respectively).

**Fig 4:**
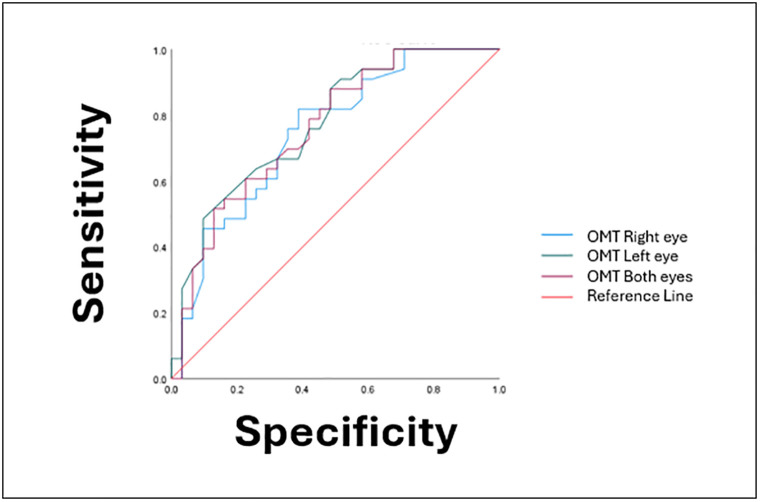
ROC curve for OMT frequency differentiating PwPD from HCs.

To assess diagnostic value of OMT assessment in PwPD, ROC curve analysis and AUC was performed (see Fig 4, Table 2). This was performed for PwPD and HC, and OMT had an AUC of 0.745-0.773.

**Table 2:** OMT frequency differentiation of PwPD from HCs.

	AUC	Cut-off (Hz)	Sensitivity	1-Specificity
**Average OMT Frequency Right eye**	.745	68.58	.818	.387
**Average OMT Frequency Left eye**	.773	65.50	.636	.258
**Average OMT Frequency Both eyes**	.760	65.13	.606	.226

AUC = Area under the curve, Hz = Hertz, OMT = ocular microtremor.

#### 3.3.2 Convergent validity.

Table 3 shows the relationship between OMT frequency for each eye and age, sex, disease duration and overall UPDRS motor score. Overall, OMT frequency negatively correlated with age in PwPD and HCs. OMT frequency correlated with sex in the PD group but not for HC. However, specific items on the MDS-UPDRS motor subsection in PwPD, showed lower OMT frequency in the right, left and both eyes correlated with poorer speech (item 3.1 in the MDS-UPDRS) (r = -.477, p = .005, r = -.367, p = .036, r = -.432, p = .012 respectively) and poorer facial expression (item 3.2) (r = -.521, p = .002, r = -.404, p = .020, r = -.474, p = .005 respectively). Additionally, poorer performance of pronation of the left hand (Item 3.6) correlated with lower OMT frequency in the left eye (r = -.380, p = .029). Finally, poorer gait performance (item 3.10) correlated with lower OMT frequency in the left eye (r = -.486, p = 004), and in both eyes (r = -.414, p = .017).

**Table 3:** Relationship between average OMT frequency and clinical measures.

r (p)	Age (years)	Sex	Disease Duration (years)	UPDRS	H &Y	FES-I	nFOGQ	LEDD
**HC**								
Overall average OMT Right eye correlation	**-0.53 (.0.002)**	0.24 (0.19)	–	–	–	-0.06 (0.77)	–	–
Overall average OMT left eye correlation	**-0.53 (0.002)**	0.24 (0.20)	–	–	–	-0.07 (0.74)	–	–
Overall average OMT both eyes correlation	**-0.53 (0.002)**	0.24 (0.19)	–	–	–	-0.07 (0.75)	–	–
**PwPD**								
Overall average OMT Right eye correlation	**-0.40 (0.02)**	**0.27 (0.04)**	-0.01 (0.96)	-0.13 (0.47)	-0.14 (0.42)	-0.16 (0.45)	- 0.07 (0.69)	0.29 (0.10)
Overall average OMT left eye correlation	-0.29 (0.10)	**0.38 (0.30)**	-0.02 (0.92)	-0.25 (0.16)	-0.25 (0.21)	-0.26 (0.21)	-0.20 (0.27)	0.14 (0.44)
Overall average OMT both eyes correlation	**-0.36 (0.04)**	**0.39 (0.03)**	-0.14 (0.94)	-0.19 (0.29)	-0.19 (0.30)	-0.21 (0.31)	-0.13 (0.46)	0.23 (0.20)

SD = Standard deviation, UPDRS = MDS Unified Parkinson’s Disease Rating Scale III, H&Y = Hoehn & Yahr Stage, FES-I= Falls Efficacy Scale – International, nFOGQ = new Freezing of Gait Questionnaire, LEDD = Levodopa Equivalent Daily Dose, HC = Healthy Control, PwPD = People with Parkinson’s Disease, OMT = ocular microtremor, r = correlation value, p = significance value.

#### 3.3.3 OMT response to a known intervention.

Table 1 shows the difference in OMT frequency (Hz) when ‘OFF’ compared to ‘ON’ anti-Parkinson’s medication. There were no significant differences in OMT readings when participants were ‘OFF’ and ‘ON’ medication, this was consistent across right eye, left eye and both eye readings.

## 4 Discussion

This pilot study aimed to examine analytical and clinical validation of OMT measurement via the novel iTremor ONE technology in PwPD. In line with our hypotheses, findings showed that OMT measurement via iTremor ONE was analytically and clinically valid in PwPD, with reliable measurement, an ability to discriminate groups, and relationships between OMT and specific motor features. However, contrary to our hypothesis there was no change in OMT with dopaminergic medication in PwPD.

### 4.1 Analytical validation

Our findings showed that the OMT measurement using iTremor ONE was robust, with excellent (ICC > 0.9) agreement for OMT frequency (Hz) detection. This level of accuracy in OMT assessment has not yet been demonstrated in wider literature using other techniques [18], perhaps suggesting greater accuracy with the novel laser-speckle technique compared to previously employed OMT detection techniques. Analytical validation of novel techniques for measuring digital biomarkers is essential for the development of appropriate and scientifically robust biomarkers in line with regulatory boards and quality management systems [48].

While reliable there were very subtle non-significant differences seen in OMT frequency between the first and second assessment that could be due to several factors. For instance, there is likely a learned effect with participant familiarisation with the researcher, device, and protocol. Other areas for variance include day-to-day fluctuations such as regarding hours of sleep, stress and caffeine intake which were not controlled

for in this study. A recent study using the Piezoelectric method in healthy adults looked at the effect of caffeine on OMT frequency [49]. Authors found that a small but significant increase in OMT frequency after caffeine intake that was still present up to 90 minutes after consumption. PwPD could have further fluctuations related to medication, fatigue, and general variability of disease, particularly in people who experience distinct ‘ON’ and ‘OFF’ periods [50–52]. Despite this, the difference in readings between assessments was small, i.e., between 1.13-1.72Hz which could be considered normal variation (and represent only 1.74-2.69% of the average measures). This would need to be investigated in a larger population to establish the expected day-to-day variation in OMT frequency.

Regarding feasibility of measuring OMT using iTremor ONE, no adverse events occurred, and no participants withdrew from the study at any stage. Only minor and occasional factors impacted ability to obtain OMT measurements (i.e., blinking, eye movement, eye lid or lash blocking reading etc.), but with a repeated measurement within session there was no data loss.

### 4.2 Clinical validation

#### 4.2.1 Comparison with controls and group differentiation.

Eye movements, such as OMT, may be useful as a diagnostic tool for differentiating groups, such as those with more severe disease or other neurological impairment. For example, vertical gaze palsy has been used to differentiate between PD and Progressive supranuclear palsy [53]. Our findings show that OMT has good (AUC 0.75-0.77) discriminative ability in differentiating between PwPD and HCs. The large effect sizes observed (Cohen’s d = 0.88-1.09) suggest that the difference in OMT frequency between PwPD and HC is not only statistically significant but also of substantial magnitude, supporting the potential clinical relevance of OMT as a discriminative measure. OMT was able to differentiate groups but there was overlap between group scores that suggest OMT frequency may provide a supporting measure for diagnosis or screening rather than a standalone measure (Fig 4). Rather than act as a diagnostic tool itself, measurement of OMT could be a part of a diagnostic testing battery to help clinicians in their decision making, particularly in ambiguous cases where symptoms overlap with other conditions. However, further studies are needed to determine this. Overall, across PwPD and HCs the OMT frequency ranged from ~63–70Hz, which is lower than values found in a previous study (e.g., ~ 68–88Hz [29]) and may reflect technical and clinical differences in the studies. For example, the iTremor ONE may be more accurate than previous OMT measurement tools (e.g., piezoelectric) [18] and the only other OMT study in PwPD offers little insight into the demographics or clinical features (i.e., only age is reported) of the groups being studied [29], which limits direct comparison.

#### 4.2.2 Comparison with traditional clinical rating scales.

Increased age related to lower OMT frequency in both PwPD and HCs, which is supported by previous research that has shown that OMT frequency declines over 60 years of age [12]. This is possibly due to change in eye movement pattern in older age as a result of various changes in neurological and physiological functioning [54]. Age related change in ocular functioning can be attributed to neurological changes such as reduced neuronal density or decreased neurotransmitter efficiency in frontal eye fields, the superior colliculus, and brainstem structures which all play key roles in oculomotor control [55]. Decreased neurotransmitter efficiency associated with age involves both the cholinergic and dopaminergic systems, which are crucial for control of eye movements such as saccade initiation and control of smooth pursuit [55]. These factors can interact and compound each other with ageing and there can be vast individual differences from person to person.

Eye movement deficits in PwPD have shown further decline with disease progression. For example, anomalies in voluntary saccadic eye movements are seen in the initial stages of PD and deficits progress to impair reflexive saccades in later stages [56]. However, overall disease severity (UPDRS-III or H&Y stage) did not correlate with OMT frequency, which was unexpected. This could suggest that the UPDRS-III or H&Y scale don’t possess the sensitivity to universally assess all of the nuances associated with PD symptomatology and symptom fluctuation, possibly due to their limited scoring (ceiling/floor effects) and focus on particular motor symptoms (i.e., UPDRS-III on tremor or bradykinesia, and H&Y scale on side of motor issue and balance) [51,52,57,58]. Additionally, the UPDRS-III and H&Y staging rely on expert clinical judgement and consequently, subjectivity. Conversely, OMT frequency is objectively measured on a continuous scale, and the traditional scales may not reflect the same underlying activity that OMT measures. When broken down into each item of the UPDRS-III, correlations arose for speech, facial expression, upper limb movement and gait, which suggests shared underlying neural processes for these aspects. For example, speech, gait, and facial expression are all early signs of PD that are linked to cognitive function and the cholinergic system, particularly involving brainstem cholinergic nuclei such as the pedunculopontine nucleus, therefore OMT may be underpinned by similar mechanisms [33,59–62]. Gait is also mediated by brainstem circuitry [63]. Similarly, it has been hypothesised that OMT generation occurs due to constant activity of the extraocular muscles stimulated by impulses from oculomotor neurons found in the brainstem [16,25,23,64,65]. These oculomotor neurons are located in the motor nuclei of the brainstem tegmentum, which lie adjacent to and are influenced by the reticular formation [16]. However, the specific brainstem nuclei, pathways, and neurotransmitter systems mediating OMT remain uncertain. While we found selective relationships with specific demographic and clinical outcomes, further research is required to establish findings and determine the neurotransmitters or neural mechanisms underlying OMT frequency in PwPD.

#### 4.2.3 Response to a known intervention.

No differences were seen in OMT frequency when tested ‘OFF’ (using a 12hr washout period) compared to ‘ON’ (1hr after consumption) anti-Parkinson’s medication, which could suggest that other neurotransmitters may be involved (e.g., cholinergic, serotonin etc.) [66]. A previous paper using the piezoelectric strain gauge technique suggests that OMT frequency in PD decreased when ‘OFF’ medication [29]. However, within this previous study, the same patients were not tested ‘ON’ and then ‘OFF’ dopaminergic medication. Instead, PwPD were categorised into groups of ‘ON’ or ‘OFF’ based on the bradykinesia section of the Webster Scale rather than a medication washout period (i.e., if the clinician deemed that someone moved more slowly, they were placed into the ‘OFF’ group irrespective of medication intake), which is subjective and reflects disease severity rather than the effect of medication on OMT. Although, within this previous study there was also one patient where dopaminergic medication was withdrawn for 48 hours due to a secondary hypertension issue, and OMT was reduced (24hours ON: 78.5Hz, 48hours OFF 66.5Hz) [29]. However, this is a single patient that had other medical issues being actively investigated and both their ‘ON’ and ‘OFF’ OMT frequency recordings were greater than the current studies PwPD and healthy older adult readings (range ~63–70Hz, Table 1), which may indicate that this one patient is not representative of PwPD.

In the present study, a standard 12 Hour washout period was used, and no difference in OMT was detected. While the previous OMT study in PwPD had a longer washout period due to an ongoing medical investigation of the patient involved [29], longer washout periods in those not required to withdraw for other medical reasons come with challenges (e.g., patient safety and falls risk). Lack of differences between ‘ON’ and ‘OFF’ dopaminergic medication in PwPD may be due to OMT not being underpinned by the dopaminergic system, and that other neurotransmitters such as acetylcholine, GABA, or Serotonin are involved [67]. There is evidence for this in eye movement outcomes such as saccades and pupil response with involvement of both the cholinergic and serotonin systems [66,68]. Additionally, cholinergic deficits lead to impairments in other involuntary eye movements such as pupillometry [68]. Therefore, further investigation is required to understand the neural mechanisms underpinning OMT in PwPD, which will inform the use of these measures in clinical trials and healthcare.

### 4.3 Study strengths and limitations

This pilot study had several strengths and limitations. This is the first study to assess OMT using a non-invasive technique in PwPD and HC. This study also assessed a range of feasibility, validity, and reliability measures. However, the study consisted of a homogeneous mild-to-moderate PD cohort (H&Y stage II-III) with no stratification by PD phenotype (e.g., tremor-dominant vs. akinetic-rigid subtypes) or disease duration. Therefore, findings don’t necessarily reflect other stages of disease, phenotypic subtypes, or those with comorbidities such as cognitive impairment. Future studies should recruit larger, more heterogeneous samples across a broader range of disease severity and phenotypic subtypes to improve generalisability. Furthermore, this study was conducted within the North East of England, which may limit generalisability to more diverse populations and geographic regions. Additionally, whilst key confounding factors were controlled, the potential for unmeasured confounding cannot be fully excluded. Specifically, factors such as caffeine intake, sleep quality, and home environment were not formally recorded and should be controlled for in future studies. Analyses were also limited to univariate correlational and group comparison approaches. Future studies should employ multivariable modelling to better account for potential confounding variables. Given that this is the first study to measure OMT non-invasively in PD, we encourage readers to interpret with caution and strongly recommend future confirmatory studies in larger cohorts to confirm findings. Future studies should also explore comparison of OMT with established PD biomarkers such as DaTscan imaging, blood-based biomarkers or other eye movement biomarkers such as saccadic eye movements to improve understanding of neurological underpinnings of OMT control.

### 4.4 Conclusion

This pilot study was the first to investigate the validity of non-invasive measurement of OMT in PwPD and HCs. We provide analytical and clinical validation evidence for the iTremor ONE in PwPD. Specifically, OMT frequency was reliably measured in PwPD, and had a good ability to differentiate between PwPD and HCs. OMT frequency related to age in both PwPD and HCs and further related to selective aspects of disease severity in PwPD. However, OMT frequency did not change with dopaminergic medication in PwPD. Future studies are required to understand the neural mechanisms underlying OMT frequency in PwPD, which will inform use of this digital biomarker in clinical trials or healthcare settings.

## Supporting information

S1 Table Missing Cases.
(DOCX)

S1 Fig Bland Altman Plots.
(DOCX)
