# Supplementary material for: Digital measurement of ocular microtremor in Parkinson’s Disease: Analytical and clinical validation
Source: PLOS Digit Health. 2026 Jun 18;5(6):e0001439. doi: 10.1371/journal.pdig.0001439 (PMC13278424; doi:10.1371/journal.pdig.0001439)
Supplement: S1 Table — Missing Cases. (DOCX) [file pdig.0001439.s001.docx]

**S2 Table: Missing cases**

|  |  | **TMT A** | **TMT B** | **CLOX1** | **CLOX2** | **nFOG** | **FES-1** | **JLO** | **Visual Acuity(Both eyes)** | **Contrast Sensitivity (Both Eyes)** | **OMT_R1** | **OMT_R2** | **OMT_R3** | **OMT_RAV** | **OMT_L1** | **OMT_L2** | **OMT_L3** | **OMT_LAV** | **OMT_BAV** |
| --- | --- | --- | --- | --- | --- | --- | --- | --- | --- | --- | --- | --- | --- | --- | --- | --- | --- | --- | --- |
| **HC** | |  |  |  |  |  |  |  |  |  |  |  |  |  |  |  |  |  |  |
| **N** | **Valid** | 30 | 29 | 14 | 14 | 0 | 24 | 22 | 23 | 23 | 31 | 31 | 31 | 31 | 31 | 31 | 31 | 31 | 31 |
|  | **Missing** | 1 | 2 | 17 | 17 | 31 | 7 | 9 | 8 | 8 | 0 | 0 | 0 | 0 | 0 | 0 | 0 | 0 | 0 |
| **PwPD** |  |  |  |  |  |  |  |  |  |  |  |  |  |  |  |  |  |  |  |
| **N** | **Valid** | 23 | 22 | 14 | 14 | 33 | 26 | 19 | 25 | 25 | 33 | 33 | 33 | 33 | 33 | 32 | 30 | 33 | 33 |
|  | **Missing** | 10 | 11 | 19 | 19 | 0 | 7 | 14 | 8 | 8 | 0 | 0 | 0 | 0 | 0 | 1 | 3 | 0 | 0 |

|  |  |  |  |  |  |  |  |  |  |  |  |  |  |  |  |  |  |  |  |
| --- | --- | --- | --- | --- | --- | --- | --- | --- | --- | --- | --- | --- | --- | --- | --- | --- | --- | --- | --- |
|  | |  |  |  |  |  |  |  |  |  |  |  |  |  |  |  |  |  |  |
|  |  |  |  |  |  |  |  |  |  |  |  |  |  |  |  |  |  |  |  |
|  |  |  |  |  |  |  |  |  |  |  |  |  |  |  |  |  |  |  |  |
|  |  |  |  |  |  |  |  |  |  |  |  |  |  |  |  |  |  |  |  |
|  |  |  |  |  |  |  |  |  |  |  |  |  |  |  |  |  |  |  |  |
|  |  |  |  |  |  |  |  |  |  |  |  |  |  |  |  |  |  |  |  |

*[TMTA/B = Trail making task, CLOX1/2 = Royall’s clock drawing task, , nFOGQ = new Freezing of Gait Questionnaire, JLO = Judgment of Line Orientation task, OMT_R 1/2/3 = 1^st^, 2^nd^, and 3^rd^ OMT reading for the Right eye, OMT_RAV = average OMT in the right eye, OMT_L 1/2/3 = first, second and third OMT reading for the Left eye, OMT_LAV = average OMT in the left eye, OMT_BAV = average OMT across both eyes, HC = Healthy controls, N = number, PwPD = People with Parkinson’s Disease.]*
