# Supplementary material for: Digital measurement of ocular microtremor in Parkinson’s Disease: Analytical and clinical validation
Source: PLOS Digit Health. 2026 Jun 18;5(6):e0001439. doi: 10.1371/journal.pdig.0001439 (PMC13278424; doi:10.1371/journal.pdig.0001439)

**S1 Fig: Bland-Altman Plots**

Bland-Altman Plot – OMT (Hz) in the Right Eye:


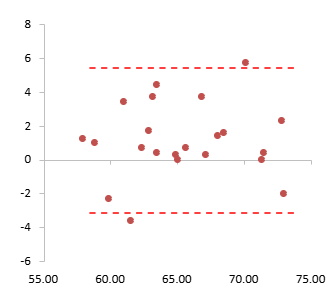


Bland-Altman Plot – OMT (Hz) in the Left Eye:
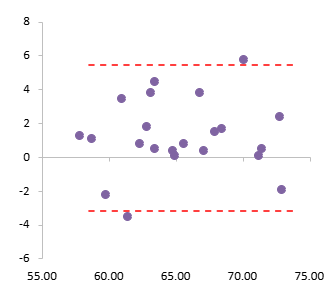




Bland-Altman Plot – OMT (Hz) in Both Eyes:


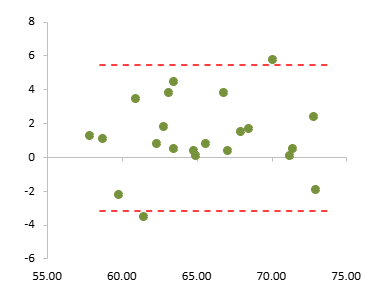

Supplement: S1 Fig — Bland Altman Plots. (DOCX) [file pdig.0001439.s002.docx]
